# Circadian Disruption and the Risk of Developing Obesity

**DOI:** 10.1007/s13679-025-00610-6

**Published:** 2025-02-13

**Authors:** Hélène Duez, Bart Staels

**Affiliations:** Univ. Lille, INSERM, CHU Lille, Institut Pasteur de Lille, U1011 - EGID, F-59000 Lille, France

**Keywords:** Obesity, Shift-work, Social jetlag, Circadian misalignment, Time-restricted eating/feeding

## Abstract

**Purpose of the Review:**

This review summarizes recent evidence for a role of the clock in adipose tissue physiology and the impact of circadian desynchrony on the development of obesity.

**Recent Findings:**

Circadian disruptions due to shift work, late time eating and nighttime light exposure are associated with obesity and its metabolic and cardiovascular consequences. Studies in mice harboring tissue-specific gain/loss of function mutations in clock genes revealed that the circadian clock acts on multiple pathways to control adipogenesis, lipogenesis/lipolysis and thermogenesis. Time-restricted eating (TRE), aligning feeding with the active period to restore clock function, represents a promising strategy to curb obesity.

**Summary:**

While TRE has shown clear benefits, especially in participants at higher cardiometabolic risk, current studies are limited in size and duration. Larger, well-controlled studies are warranted to conclusively assess the effects of TRE in relation to the metabolic status and gender. Field studies in shift-workers, comparing permanent night shift versus rotating shifts, are also necessary to identify the optimal time window for TRE.

## Introduction

With overweight and obesity affecting more than half of the adult population in 2022 and more than a quarter of children and adolescents worldwide [1], obesity has become a major public health problem and an important driver of the development of metabolic (type 2 diabetes, metabolic dysfunction-associated steatotic liver disease [MASLD]) and cardiovascular complications. Although excess food intake and physical inactivity are traditional risk factors of the growing obesity epidemic, circadian misalignment and concurrent sleep restriction have emerged as a novel contributing factor.

“24 hours a day, seven days a week” (24/7) modern lifestyles have dramatically changed the daily rhythm of life. Food intake and light exposure are no longer restricted to day (light) time, and approximately 25% of the working population is night/shift working. These changes result in circadian misalignment, ie a mismatch between socially- or economically-driven behavioral rhythms and the internal circadian system, and concurrent sleep restriction and mismatched timing of eating. However, circadian misalignment and shortened sleep duration reduces energy expenditure, alters the levels of appetite hormones and promotes unhealthier food choices, and are associated with adverse health outcomes including an increased risk of developing obesity [2–6]. While allowing major advances in modern-day society, mistimed modern electric lighting and late exposure to electronic devices is a contributing factor to delayed and reduced sleep, and an important factor of chronic social jetlag [7]. Obesity is associated with late night eating patterns and extended feeding windows, whereas aligning meal timing with the daylight/early active period efficiently reduces adiposity in mice as well as in humans [8, 9]. Thus, a better understanding of the cellular and molecular mechanisms linking the circadian clockwork with energy balance and adipose tissue physiology is necessary to combat obesity and related complications. In this review, we summarize the recent evidence linking circadian misalignment and the risk of obesity in mice and humans, review the cellular and molecular mechanisms focusing on preclinical mouse studies and discuss the potential of TRE to restore robust rhythmicity and mitigate obesity in man.

## Current View of the Mammalian Molecular Clock and Temporal Architecture of Physiology

**Fig. 1:**
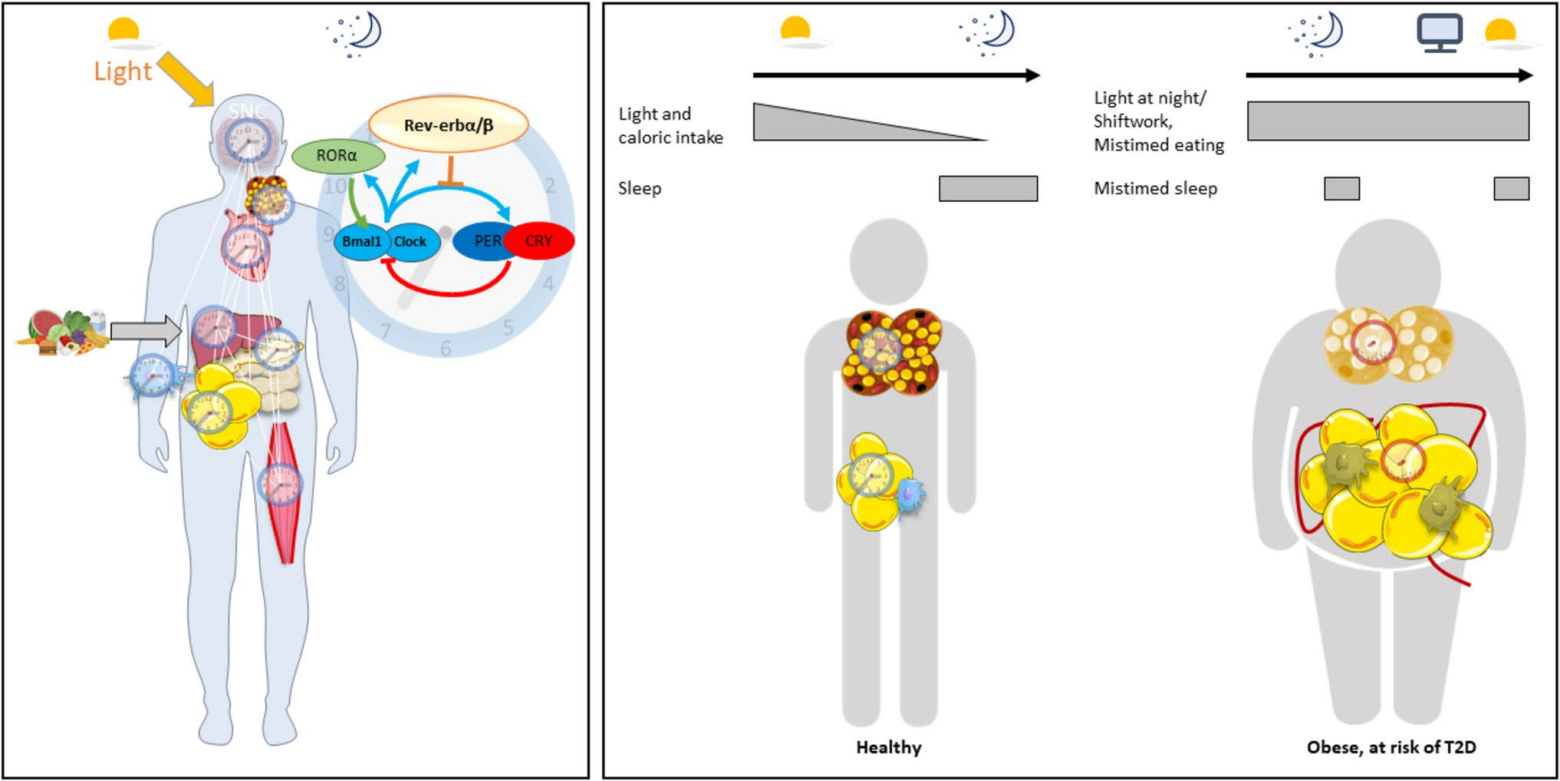
**Left:** The biological clock consists of a central clock located in the suprachiasmatic nucleus in the hypothalamus and synchronised by the day/night cycles. The central clock tunes peripheral clocks located in all cells/organs. The synchronisation of peripheral clocks can also be influenced by other stimuli, such as feeding/fasting cycles. The molecular clock consists of transcription–translation feedback loops. The transcription factors BMAL1/CLOCK induce the expression of the negative regulators Period (PER) and Cryptochrome (CRY). In turn, the PER/CRY heterodimer inhibits the transcriptional activity of BMAL1/CLOCK. Once PER and CRY levels are sufficiently low, a new cycle starts. CLOCK/BMAL1 induce the expression of the nuclear receptors Rev-erbα/β and retinoid-related orphan receptor α (RORα). Rev-erbs and RORα repress or activate, respectively, Bmal1 and Clock. **Right**: Coordinated metabolic processes orchestrated by peripheral clocks are essential to ensure metabolic heath and weight management. By contrast, peripheral clock desynchrony due to shift work, late eating patterns, exposure to light at night, results in metabolic dysfunctions and obesity

Circadian rhythms evolved to anticipate and adapt to daily variations in the environment. As an example, metabolic needs are different during the active and the rest periods. Circadian clocks are present in every single cell of the body and orchestrate gene expression, protein level and activity, behavioural and metabolic programming over the 24-h light–dark cycle so that feeding, energy processing and activity coincide with wakefulness, while fasting and inactivity are concurrent with sleep. At the molecular level, the clock is a cell-autonomous autoregulatory network composed of core transcription factors organized in several transcriptional loops: BMAL1 (ARNTL) and CLOCK heterodimerize to positively regulate the transcription of Per and Cry which, in turn, repress their own BMAL1/CLOCK-driven transcription (Fig. 1). In a second loop, BMAL1/CLOCK controls the transcription of RORs and Rev-erbs which, in turn, rhythmically activate or repress, respectively, Clock and Bmal1 transcription [10]. The core clock network regulates downstream output pathways, generating substantial rhythmicity in transcription (~5–20% of genes expressed in any particular cell or tissue are rhythmic). Translation and post-translational modifications such as phosphorylation and acetylation, as well as metabolite abundance are also under the control of the biological clock and display daily fluctuations [11–14].

Interestingly, the vast majority of circadian programs is organ-specific and allows an intricate rhythmic orchestration of the pathways necessary for organ function. The central clock, located in the suprachiasmatic nuclei of the hypothalamus, is set by light. Peripheral clocks receive input from the central clock to ensure coordinated systemic “resonance” through hormonal and neuronal cues [15, 16]. Peripheral clocks integrate additional time cues from food, which is essential to gate metabolic processes to the optimal time-window. Feeding mice exclusively during their resting phase causes a progressive phase shift of most peripheral clocks which adjust more or less quickly to the new feeding schedule [17–19], while the central clock remains phase-locked to the day/night cycle. This has major implications in the development of circadian desynchrony due to mistimed food intake, as is often the case for shift workers, while time restriction of food intake to appropriate times enforces robust cycling [9]. In the liver, insulin, which is secreted in response to feeding/fasting cycles, resets the clock [20–22]. Interestingly, the adipose tissue clock is phase-shifted by delaying food access also in humans [23]. Additionally, peripheral tissue clocks sense nutrients via the nicotinamide phosphoribosyltransferase (NAMPT), Sirtuin 1 (SIRT1), AMPK and mTOR pathways as well as by modulating nuclear receptor activities which all impinge on clock components [24–26].

## Evidence of Circadian Oscillations in White Adipose Tissue Physiology Altered in Obesity

Excess calories from food are stored as triglycerides (TGs) in white adipocytes, whereas thermogenic, mitochondria-rich brown adipocytes dissipate energy. Interestingly, white and brown adipose tissue possess molecular clocks that orchestrate rhythmic gene expression to adapt to environmental stimuli and control energy intake and use during the day/night (feeding/fasting) cycle. 4% of the genes (corresponding to 856 transcripts) in white adipose tissue (WAT) exhibit a circadian expression profile, and 8% are cycling in brown adipose tissue (BAT) [27]. Similar transcriptomic rhythmicity (approximately 2% of the transcriptome) was found in human subcutaneous WAT in healthy young male subjects, and robust oscillations were observed in the expression of clock genes and genes related to metabolism and gene expression regulation (DNA/RNA binding, transcription factor and co-factor binding) [28].

In stark contrast, clock function is considerably attenuated in obesity. In a landmark study, Kohsaka and colleagues reported that feeding mice a high fat diet (HFD) led to disrupted cycling of clock genes and metabolic genes in many tissues, including WAT, attenuated locomotor activity and mistimed food intake, with an increased amount of food consumed in the light (resting) period and a decrease in food intake during the dark period [29]. In addition, diet-induced obesity led to a relocation of BMAL1 DNA occupancy, thereby altering the rhythmic transcription of numerous genes involved in metabolic pathways, inflammation and matrix remodelling [30]. Similarly, Bmal1 expression was strongly reduced in WAT from genetically (ob/ob) obese mice [31], along with lower methionine and glutamine levels. Interestingly, reduced glutamine-to-glutamate ratios are associated with obesity-related insulin resistance in mice and humans [32]. Moreover, together with altered feeding behaviors, flattened rhythms of Rev-erbα, Per1 and Cry1 gene expression were observed in WAT from ob/ob mice [31].

In humans, the circadian clock function is also impaired in omental and subcutaneous WAT of obese insulin resistant/diabetic patients coincidently with higher levels of inflammation and fibrosis markers compared to lean subjects [30, 33]. Interestingly, this was associated with a loss of rhythmic genes, notably in pathways regulating lipolysis such as the PPARα, AMPK and cAMP-mediated signalling pathways. This may explain the reduced amplitude in non-esterified fatty acids (NEFA) plasma levels, especially the higher trough in post-prandial NEFAs observed in obese diabetic individuals. Another study investigated whether weight loss in overweight subjects affects the expression of clock genes in human WAT. They found that an improved metabolic profile (improved lipids, lower fasting glucose, reduced BMI) along with weight loss was associated with significant increases in PER2 and REV-ERBα gene expression in subcutaneous fat, together with changes in lipid-related (LPL, Fatty Acid Synthase, NAMPT) and inflammatory genes (NLRP3, TLRs) [34].

## Evidence of Circadian Control of Brown Adipose Tissue Activity in Mice and Humans

Whereas WAT stores excess energy, brown fat is thermogenic, dissipating energy in the form of heat. One pathway involves mitochondrial uncoupling via the activity of uncoupling protein 1 (UCP1). The presence of BAT in humans correlates with lower body weight, and cold-activated BAT increases glucose and lipid disposal, suggesting that activation of BAT may possibly have a beneficial impact on obesity and cardiometabolic outcomes [35, 36]. BAT glucose uptake and activity oscillate during the light/dark cycle in mice, resulting in circadian rhythms in body temperature, being highest during the awake active period and lowest while asleep [37, 38]. Strikingly, extended exposure of mice to day light (> 16h/24h) for 5 weeks tended to diminish BAT activity, resulting in elevated body fat mass [39]. In mouse BAT, rhythmic genes are encoding proteins involved in the regulation of adipogenesis and lipogenesis, such as ATP citrate lyase and glucokinase, which are maximally expressed around ZT18-ZT0 (ie late active and onset of the sleep phase), while genes involved in lipid catabolism, such as lipoprotein lipase (Lpl) and patatin-like phospholipase domain containing 2 (Pnpla2, also known as ATGL), peak at ZT8-ZT14 (ie around awakening), concurrent with increasing thermogenesis at the beginning of the active phase [40]. It has been suggested that intracellular lipolysis at the end of the inactive phase serves to supply BAT with thermogenic substrates, followed by replenishment by fatty acids (FA) taken up from triglyceride-rich lipoproteins (TRLs) after lipolysis by LPL and subsequent lipogenesis and storage as TGs around the onset of the active (feeding) phase [40]. Consistently, FA uptake from circulating TRLs by BAT is rhythmic, being higher during the early active phase, coinciding with elevated post-prandial clearance of lipids [41].

A recent study in mice investigating the impact of day vs nighttime feeding schedules on adipose tissue clock gene expression revealed that, unlike the liver and visceral WAT, but similar to the hypothalamus, the BAT circadian clock is insensitive to feeding time [42]. In WAT, daytime feeding shifted patterns of rhythmic gene expression with a decrease in the amplitude of some clock genes (Rev-erbα and Per2). As a result, gene expression profiles of day vs night time eaters were anti-phasic in WAT. By contrast, in BAT some, but not all genes were shifted in response to daytime feeding. Indeed, neither the phase nor the amplitude of clock genes or genes involved in thermogenesis (*Ucp1*) were affected by daytime feeding in BAT, while other genes, such as *Ucp2* or *Leptin,* were shifted, pointing to an internal rhythmic misalignment in this tissue when mice are fed at irregular hours [42].

## Genetic and Environmental Clock Dysfunction Leads to Obesity in Mice and Humans

### Lessons From Mouse Studies

Chronic clock dysregulation, induced by light exposure conditions mimicking rotating shiftwork, resulted in enlarged adipocytes, increased macrophage infiltration with the presence of typical crown-like structures around apoptotic adipocytes, and fibrosis in both visceral and subcutaneous WAT in mice, which was associated with impaired insulin signaling. Transcriptomic analyses revealed consistent up-regulation of inflammatory and adipogenic pathways, and disruption of normal time-of-the-day-dependent gene regulation [43]. In a seminal study assessing the impact of genetic perturbation of the *clock* gene, Turek and colleagues found increased body weight and greatly attenuated diurnal feeding rhythms and hyperphagia, together with hyperleptinemia, hyperlipidemia, hepatic steatosis, hyperglycemia and insulin resistance in homozygous C*lock* mutant mice fed a HFD, pointing to an important role of the circadian clock in the control of mammalian energy balance [44]. In line, genetic models of clock disruption generally develop obesity and adverse metabolic consequences. For instance, Cry1/Cry2 double knockout (Cry1/2 DKO) mice become more obese, while eating less than controls when fed a HFD [45]. Increased fat mass was associated with larger adipocytes and up-regulated lipogenic gene expression [45]. CRY1 was also reported to induce adipogenesis via Wnt/β-catenin signaling [46]. Furthermore, Cry1/2 DKO mice displayed small lipid droplets in interscapular BAT. Accordingly, adipogenesis was impaired and expression of BAT-selective genes and regulators, such as Ucp1, Cidea, Pparɣ, Adipoq and Fabp4, was suppressed in Cry1/2 DKO brown adipocytes, revealing an important role of CRY1 and/or CRY2 also in BAT differentiation [47].

Other clock genes also play key roles in adipose tissue functions and energy balance. Bmal1-deficient fibroblasts failed to differentiate into adipocytes [48], while others reported increased adipogenic differentiation and adipocyte hypertrophy in whole-body Bmal1-deficient mice [49], in agreement with the observed higher body weight and greater adiposity in whole-body and adipocyte-specific Bmal1-deficient mice [50]. Adipocyte (white and brown)-specific Bmal1-deficient mice exhibit increased body weights when fed a regular diet, and become more obese when fed a HFD compared to controls, but without sign of increased inflammation nor fibrosis [50]. These mice were protected from HFD-induced glucose intolerance and insulin resistance [50, 51]. Diminished Bmal1 expression in macrophages due to the use of the ap2 Cre driver may explain the absence of inflammation and impact on insulin sensitivity [50]. Since Bmal1 drives Rev-erbα expression, decreased adipocyte Rev-erbα expression in adipocyte-specific Bmal1-deficient mice may contribute to their phenotype. Rev-erbα expression increases during adipocyte differentiation and concomitantly promotes adipogenesis of mouse fibroblasts, whereas subsequent reduction in its protein levels may be necessary for complete differentiation into mature adipocytes [52, 53]. This activity, together with its role in lipogenesis (increased glyceroneogenesis and up-regulation of fatty acid synthesis), may contribute to the increased adiposity observed in whole-body Rev-erbα-deficient mice ([53, 54] and our unpublished observations). Integration of transcriptomic and cistromic data only identified a limited number of direct Rev-erbα target genes in WAT, including clock and collagen biosynthesis genes, when mice are fed a regular diet. Yet, challenging the mice with a HFD revealed a more pervasive role of Rev-erbα which extended to the regulation of lipid and mitochondrial pathways in obese adipocyte-specific Rev-erbα-deficient mice, yet without inflammation, fibrosis nor deterioration of glucose tolerance and insulin sensitivity [55]. The increased adiposity of adipocyte Rev-erbα-deficient mice, when fed the obesogenic diet, reflects an increased storage and adipose tissue expansion. Thus, it was suggested that adipose tissue Rev-erbα serves to control metabolic pathways and adipose tissue expansion in obesogenic conditions. RORα, which controls transcription through binding to similar DNA response elements, also controls adipogenesis in vitro [56]. Studies in adipocyte-specific mice are still awaited to define the precise physiological role of RORα in this cell type.

In the above studies, neither whole body Bmal1-deficiency nor adipocyte Rev-erbα deletion altered the expression of thermogenic genes (Pgc1α, Ucp1) in BAT under regular temperature conditions [50, 55]. By contrast, diurnal oscillations in BAT glucose uptake were abolished in Rev-erbα-deficient mice placed at thermoneutrality. Glucose uptake remained high during the day and night time. This was paralleled by a loss of oscillations in body temperature and BAT thermogenic activity which remained high during the light phase, leading to improved tolerance to cold during the light phase. Genetic loss of Rev-erbα abolished circadian rhythms of Ucp1 expression [38]. Human brown fat tissue explants from young healthy volunteers take up glucose, oxidize fatty acids and display a thermogenic activity in a rhythmic manner [57, 58]. In synchronized primary human brown adipocytes, Ucp1 and Glut 4 expression are anti-phasic to Rev-erbα, suggesting a similar regulation of Ucp1 gene expression by Rev-erbα in humans [57].

Conversely, BAT-specific Bmal1 deletion in mice resulted in lower Rev-erbα and Per1 expression, and a loss of Ucp1 rhythmicity with, generally, higher levels across the 24h cycle similar to what is observed in Rev-erbα KO mice [59]. However, BAT thermogenesis is reduced upon deletion of Bmal1, consistent with a higher phosphocreatine/creatine (PCr/Cr) ratio and reduced creatine cycling [59], which is known to drive Ucp1-independent thermogenesis [60]. Interestingly, brown adipocytes were larger and FA utilization, measured by indirect calorimetry, and circadian expression of lipid-related genes were impaired in BAT Bmal1 KO mice, in line with a more HFD-induced obese phenotype [59]. Finally, Per2 acts as a negative regulator of adipogenesis through direct binding to and inhibiting the activity of PPARɣ, a master regulator of adipogenesis, hence impeding white adipocyte differentiation [61]. By contrast, mice deficient in PER2 (global deletion) exhibit reduced FA utilization and UCP1 expression in BAT, resulting in a reduced ability to maintain temperature in response to cold exposure [37]. PER2 binds to the *Ucp1* promoter to increase its expression, but binding was lost in PPARα-deficient mice, suggesting that PER2 acts as a PPARα co-activator. Altogether these studies demonstrate that the molecular clock controls both white and brown adipocyte function and, consequently, disruption of circadian rhythms impacts fat accumulation and thermogenesis.

Adipose tissue metabolism is also controlled by infiltrated immune cells. Interestingly, Bmal1 and Per1/2 within myeloid cells impact HFD-induced body weight gain, although conflicting results were reported (accumulation of visceral WAT and BAT and inflammation upon myeloid cell-specific Bmal1 or Per1/2 deletion [62, 63] *vs* no effect on weight gain in myeloid Bmal1 deficient mice [50]. A recent study suggested that visceral WAT Treg cells control daily rhythms of WAT lipolysis, and deletion of Bmal1 in Tregs attenuated WAT lipolysis, increased adiposity and visceral fat inflammation upon HFD feeding [64]. Finally, rhythmic IL-17 producing innate ɣ/δ T cells maintain adipose tissue homeostasis through proper circadian control of *de novo* lipogenesis, in particular in BAT [65].

### Shiftwork and Social Jetlag Increase the Risk of Obesity

About one quarter of the active population in the USA and Europe is shift working due to the economic demand and societal pressure. Shift workers are exposed to light at night which is accompanied by a disruption of usual sleep patterns, insufficient sleep as well as altered feeding patterns with access to food during atypical hours resulting in increased snacking of carbohydrates [66]. In an in-laboratory 4-nights-shift study, the majority of rhythmic genes, not only clock genes, did not adjust to a night schedule, indicating a loss of temporal regulation of metabolic, inflammatory and other pathways [67]. Several observational studies and meta-analyses have linked circadian misalignment and reduced sleep duration in real-life shift workers or in forced desynchrony study protocols with adverse health outcomes, including an increased risk for obesity and diabetes (Table 1) [2, 3, 5, 66–70]. A number of studies reported a positive, “dose-response” relationship between the magnitude of night shift work and the incidence of overweight/obesity, which increased with both number and duration of night shifts [70]. Additionally, permanent night workers demonstrated a higher risk than rotating shift workers [70]. Milder social jetlag, defined as the shift of sleep timing between work (or school) days and free days (late awakening to compensate sleep debt), is highly prevalent in the population (up to 80%) and also thought to promote overweight/obesity, especially when food is consumed later in the day [2, 71, 72]. A recent meta-analysis of 43 observational studies in children/adolescents and adults including >230 000 subjects confirmed that social jetlag positively associates with body mass index, fat mass, percent of body fat and waist circumference [73]. Altogether, these observations indicate that living chronically against the biological clock may be a factor contributing to the obesity epidemic.

**Table 1:** Studies investigating the association between social jetlag or shift work and the risk of being overweight or obesity

Study	Population	Sample size	Results	Reference
Social jetlag				
Arab et al., 2023	Systematic review and meta-analysis of 43 studies published between 2012 and 2023. General population comprising mainly healthy individuals and also including individuals with obesity, metabolic syndrome, or prediabetes, across several countries around the world. Exclusion criteria: patients in a hospital setting or acute care, T2D patients	231 648	There was a positive association between social jetlag (SJL) and body mass index (correlation coefficient [r]: 0.12; 95%CI, 0.07, 0.17; P < 0.001; I2 = 94.99%), fat mass (r: 0.10; 95%CI, 0.05, 0.15; P < 0.001; I2 = 0.00%), fat mass index (fat mass divided by height in meter squared, β: 0.14 kg/m2; 95%CI, 0.05, 0.23; P < 0.001; I2 = 56.50%), percent of body fat (r: 0.37; 95%CI, 0.33, 0.41; P < 0.001; I2 = 96.17%), waist circumference (r: 0.15; 95%CI, 0.06, 0.24; P = 0.001; I2 = 90.83%), and the risk of having overweight/obesity (odds ratio: 1.20; 95%CI, 1.02, 1.140; P = 0.039; I2 = 98.25%).	[73]
Roenneberg et al., 2012	This is a general population-based study enrolling adolescents and adults of both sexes, primarily european participants. Exclusion critera: no regular week schedules	64 110	SJL significantly increased the probability of belonging to the group of overweight participants (OR: 3.300; 95% CI, 2.512 to 4.334).	[72]
Parsons et al., 2015	This is the New Zealand population-representative Dunedin Longitudinal Study, including participants of both sexes. Exclusion criteria: Shift-work	815	SJL was associated with metabolic syndrome (OR: 1.3; 95% CI, 1.0 to 1.6, p = 0.031) and obesity (OR: 1.2; 95% CI, 1.0 to 1.5, p = 0.045) after controlling for chronotype and sleep duration. Greater SJL is associated with a metabolically unhealthy profile among obese participants (OR = 1.4 (95% CI: 1.1–1.8), P = 0.008).	[71]
Koopman et al, 2017	The New Hoorn Study (NHS) is a population-based cohort that is representative of the general Dutch population, including participants of both sexes. Exclusion criteria: shift work and those with no information regarding sleep-related measures or metabolic syndrome, diabetes, or prediabetes.	1 585	SJL was associated with an increased prevalence of metabolic syndrome. After adjustment for sex, employment status, and educational level, prevalence ratios of 1.29 (95% CI 0.9–1.9) were observed for participants with 1–2 h SJL and 2.13 (95% CI 1.3–3.4) for participants with >2 h SJL, compared with participants with <1 h SJL in the younger <61y participants.	[74]
Shift work				
Sun et al., 2018	This systematic review and meta-analysis evaluated the associations between shift work patterns and risks of obesity in 28 studies (22 cross-sectional and 6 cohort studies).		The pooled odds ratio (OR) of night shift work was 1.23 (95% CI = 1.17–1.29) for risk of obesity/overweight. Permanent night workers showed higher risk than those rotating shift workers from 10 studies (OR = 1.43, 95% CI: 1.19–1.71 vs. OR = 1.14, 95% CI: 1.05–1.23).	[70]
Van Drongelen et al., 2011	This systematic review aims to summarize the available evidence to elucidate the effects of shift work, which includes night work, on body weight change. 8 articles were included and the number of participants in the selected studies ranged from 55 to 7.254.		Strong evidence for a crude association between shift work exposure and body weight increase was found.	[75]
Boini et al., 2022	Literature review including 33 systematic reviews, nine of them on the consequences of night-shift work on weight gain during working life.	The number of participants varies between studies from 11,537 to 311,334	A stated excess risk of being overweight at around 25% was also highlighted for shift workers overall, which could reach 38% among night-shift workers. An increased risk of obesity, estimated at 5% for night-shift workers and at 18% for rotating shift workers, was observed.	[76]
Zhang et al., 2020	Systematic review and meta-analysis of 11 eligible studies testing the association of shiftwork and the risk of obesity in nurses	74651	The pooled estimate of the risk of obesity in shift work nurses as compared to non-shift work nurses did not achieve statistical significance (OR = 1.05, 95% CI = 0.97–1.14). However, the risk of obesity was significantly higher in the sub-analysis of night-only shift work nurses (OR = 1.12, 95% CI = 1.03–1.21).	[77]

## Time Restricted Feeding/eating: a Valuable Strategy to Prevent or Reduce Obesity?

Feeding mice exclusively during the resting phase (akin to night shift eating in humans) increased their body weights even at similar amounts of consumed calories [78]. This led to the tractable concept of time-restricted feeding/eating (TRF/TRE), or in other words the ability to restore clock function and prevent or curb obesity by restricting the feeding to a time-window aligned with the active period, without intentional caloric restriction [9]. In rodents, TRF promotes/restores robust circadian and metabolic cycles, thereby mitigating obesity and metabolic dysfunction, even without a significant decrease in caloric intake [79, 80]. In mice fed a western diet, TRF improved adipogenic and thermogenic gene rhythmicity in BAT, as well as glucose and FA metabolism and oxidative phosphorylation in WAT, likely indicating better lipid handling and storage compared to isocaloric *ad libitum* feeding [81]. Moreover, in both tissues, the expression of inflammatory signalling-related genes decreased [81]. Interestingly, mistimed feeding abrogated creatine kinase B (CKB) expression and creatine abundance rhythmicity, whereas TRF to the active period enhanced BAT thermogenesis through modulation of this cycle [82]. In agreement with [59], Bmal1-deficiency decreased creatine levels and cycling, and TRF did not restore thermogenesis in this model. By contrast, over-expression of Bmal1 ameliorated metabolic fitness in diet-induced obesity [82].

Previous observation studies in humans have linked late night eating with increased BMI and body fat in healthy young males, and lower weight loss and dietary improvement of glucose control in obese adults [83, 84]. Interestingly, late night eating is associated with delayed rhythms in plasma glucose as well as expression of clock genes, notably Per 2, in adipose tissue [23]. These studies support the idea that food consumption at an inappropriate circadian time may provoke a circadian misalignment sufficient to increase the risk of obesity. Monitoring of human eating habits found that >50% of people eat >15 hours daily [85]. The first study evaluating the impact of a 16-week TRF intervention on body weight in overweight participants reported a sustained weight loss (approx. 3% of initial body mass) and less hunger at bedtime when the eating time window was reduced from >14 h to 10 h per day [85]. This finding was confirmed by several reports in overweight or obese individuals showing weight loss upon 8-10h/day TRF [86, 87]. It is noteworthy that, in these studies, TRE inadvertently reduced energy intake usually by 200–500 kcal/day, hinting at the possibility that the benefits of time-restricted feeding programs are mainly due to reductions in calorie intake. However, other studies showed an improvement in metabolic health upon TRE when isocaloric diets were used or even in the absence of weight loss [88]. TRE in insulin resistant and/or diabetic patients resulted also in weight loss, and improved cardiometabolic health (improved insulin sensitivity and beta cell function and reduced blood pressure) [88–93]. Whether the timing of the eating window (early versus late in the day) impacts weight loss and metabolic disease risk is not fully understood. In overweight men, a 9h TRE protocol improved the glycemic response to meals, but only early (8am-5pm) TRE reduced mean fasting glucose assessed by continuous glucose monitoring, whereas late TRE (12am-9pm) did not [90]. Consistently, a study comparing early morning (6am-3pm) and midday (11am-8pm) TRE in healthy non-obese individuals showed that only early TRE reduced fasting glucose, adiposity and inflammation [93]. In an in-laboratory randomized cross-over trial controlled for timing, amount and type of food, late eating and skipping breakfast increased hunger, decreased 24h plasma leptin levels and energy expenditure. Subcutaneous WAT gene expression profiles indicated better lipid storage and decreased lipolysis in late eaters [94]. Another randomized cross-over trial enrolling overweight/obese, but otherwise healthy participants comparing isoenergetic weight loss diets with morning loaded or evening loaded calories found that consumption of a larger meal early in the morning resulted in less hunger and desire to eat [95]. A recent meta-analysis concluded on the benefits of TRE in obese/overweight individuals. However, the number of participants is still relatively limited and there is a need for large scale, better controlled studies to fully assess the potential benefits of TRE in different populations [96].

TRE was recently tested in 24h shift workers. 137 firefighters, 70% of which had at least one cardiometabolic risk factor (obesity and/or high blood pressure and/or dyslipidemia and/or elevated CRP) at baseline, were enrolled in this randomized control trial which lasted 12 weeks. Daily 10h TRE decreased BMI and reduced HOMA-IR, HbA1c and blood pressure, especially in participants with higher cardiometabolic risk at baseline, despite similar reductions in caloric intake in the control and TRE arms [97]. Food intake was restricted to the light (9 AM-7 PM) period whatever their work schedule and even when working at night. Further studies looking at the impact of different time windows are warranted to determine whether alignment with the light/dark cycle or the active/sleep alternance periods would result in equal benefits.

Collectively, these studies in rodents and humans provide evidence that aligning meal timing with circadian time may sustain or amplify circadian clock signals to prevent or mitigate obesity and ensuing metabolic and cardiovascular diseases.

## Limitations and Perspectives

Most mouse studies indicate that genetic or environmental disruption of the circadian clock is linked to worsened metabolic outcomes, such as obesity and metabolic syndrome. However, discrepancies between full knockout and tissue-specific gene deletions have been observed, potentially due to compensatory effects or the impact of whole-body KO on feeding behaviours. Tissue-specific and inducible genetic studies supported by single-cell analysis are needed to clarify how the circadian system within specific cell types affects overall homeostasis in ageing animals. Challenges in these studies include the lack of specificity of cre drivers, the variety of diets used, and housing conditions. Notably, most research is conducted at a standard housing temperature of 22°C, which may lead to increased energy expenditure as mice adapt to the cold. These potential confounding factors highlight the importance of carefully designed phenotypes and the physiological effects of TRF. Mouse studies often focus on young male mice and sex differences remain under explored. Similarly, while human studies looking at the impact of jetlag or shiftwork on body weight include both men and women, potential gender differences in the effects of TRE remain largely unknown.

While TRE offers health benefits in humans, it is in several studies accompanied by reduced energy intake, suggesting that its benefits may partially arise from a reduction in caloric intake. However, metabolic improvements have been observed even with isocaloric diets or in the absence of weight loss. Despite the limited numbers of participants in most studies, these findings suggest that aligning food consumption with an individual’s biological rhythm could contribute to better weight management. Several questions remain, particularly regarding the timing and duration of the eating window. Shifting meal timing to earlier hours might result in improved insulin sensitivity, but this should be confirmed. Additionally, shorter eating windows may offer additional benefits, but it is essential to balance efficacy with feasibility (adherence). Most studies to date have focused on relatively healthy populations over short periods of time. The impact of TRE on individuals with obesity, type 2 diabetes (T2D), with or without hepatic steatohepatitis or cardiovascular complications, requires further investigation, though greater benefits might be expected in these at-risk groups.

Finally, more research is needed to confirm the benefits of TRE in shift workers and determine whether food intake should be limited to daylight hours, regardless of work schedules, and whether aligning eating patterns with the light/dark cycle or the active/sleep cycle would yield similar benefits.

## Concluding Remarks

The modern 24/7 society relies on electric light, shift-work, and constant food access, which all disturb the circadian clock, thereby increasing the risk of obesity and cardiometabolic diseases. We therefore need to understand how the clock orchestrates rhythmic metabolic processes in different tissues to ensure whole body metabolic health, and to identify novel strategies that limit cardiometabolic health risks in the population of shift workers and upon chronic social jetlag. Promising strategies focussing on preventing circadian misalignment include bright light therapy and TRE. TRE is interesting as it has usually excellent compliance due to the lack of caloric restriction. Most studies using TRE have shown clear benefits, especially in participants at higher cardiometabolic risk, but were limited in size and duration. Larger well-controlled studies are warranted to conclusively assess the effects of TRE in relation to the metabolic status and gender. In addition, there are only few studies on the impact of length and timing (early versus late) of the eating window. Finally, field studies are required to assess TRE in shift-workers comparing permanent night shift versus rotating shifts to delineate the optimal time window.

## Data Availability

No datasets were generated or analysed during the current study.

## Abbreviations

Adipoq: adiponectin encoding gene
AMPK: AMP-activated protein kinase
ap2: adipocyte protein 2
ATGL: Adipose triglyceride lipase
BAT: brown adipose tissue
BMAL1: Brain and muscle arnt-like 1
BMI: Body mass index
CI: confidence interval
Cr: creatine
CRY: cryptochrome
FA: Fatty acid
Fabp: fatty acid-binding proteins
HbA1c: hemoglobin A1c
HFD: high fat diet
HOMA-IR: Homeostatic Model Assessment for Insulin Resistance
Kcal: kilocalories
LPL: Lipoprotein lipase
MASLD: metabolic dysfunction-associated steatotic liver disease
mTOR: mammalian target of rapamycin
NAMPT: nicotinamide phosphoribosyltransferase
NEFA: non-esterified fatty acids
NLRP3: NOD-, LRR- and pyrin domain-containing protein 3
OR: odds ratio
PCr: phosphocreatine
PER: Period
PNPLA2: patatin-like phospholipase domain containing (also known as ATGL)
PPAR: Peroxisome proliferator-activated receptor
ROR: Retinoic acid-related Orphan Receptor
SIRT1: Sirtuin 1
SJL: social jetlag
TG: triglycerides
TLR: Toll-like receptor
TRE: time-restricted eating
TRF: time-restricted feeding
TRLs: triglyceride-rich lipoproteins
UCP1: uncoupling protein 1
WAT: white adipose tissue
ZT: Zeitgeber time
